# Slowing the growth of drug-resistant tumors

**DOI:** 10.7554/eLife.109866

**Published:** 2025-12-18

**Authors:** Yingyi Zhang, Qianying Lu

**Affiliations:** 1 School of Disaster and Emergency Medicine, Tianjin University Tianjin China; 2 Tianjin Key Laboratory of Disaster Medicine Technology Tianjin China

**Keywords:** cyclin dependent kinase, breast cancer, targeted therapy, drug resistance, Human, Mouse

## Abstract

Using drugs that inhibit enzymes called CDK4/6 and CDK2 extends the G1 phase of the cell cycle and helps to slow the growth of drug-resistant breast cancer.

**Related research article** Armand J, Kim S, Kim K, Son E, Kim M, Kalinsky K, Yang HW. 2025. Therapeutic benefits of maintaining CDK4/6 inhibitors and incorporating CDK2 inhibitors beyond progression in breast cancer. *eLife* **14**:RP104545. doi: 10.7554/eLife.104545.

Cancer treatments have seen significant advancements in recent decades, but resistance to therapies – even after an initial response to treatment – remains a major challenge (Sung et al., 2021). For example, metastatic breast cancer is one of the leading causes of cancer-related mortality in women, and around 30% of patients develop resistance within two years of initial treatment (O’Leary et al., 2018; Goel et al., 2018).

A key dysregulation in breast cancer involves the overactivation of certain proteins that drive cell proliferation. In healthy cells, division happens through tightly regulated phases during which DNA is duplicated before the cell splits. The G1 phase, in particular, serves as a checkpoint before DNA replication begins. Enzymes called CDK4 and CDK6 control how quickly cells grow and divide by inactivating (through phosphorylation) the retinoblastoma protein (Rb): this is a tumor suppressor that normally restrains progression through the cell cycle. This inactivation triggers the release of E2Fs, a group of transcription factors that activate genes needed for DNA replication, and promotes entry into the S phase, where DNA replication occurs. At this stage, CDK2 activity becomes progressively dominant over CDK4/6, enabling full transition into the S phase and ensuring DNA replication proceeds correctly.

In hormone-driven breast cancer, CDK4 and CDK6 are often overactive. Despite the use of drugs that inhibit these enzymes (Johnston et al., 2020), some tumors can resist treatment by activating CDK2: this suggests that targeting both CDK4/6 and CDK2 may be necessary to overcome resistance. Now, in eLife, Hee Won Yang and colleagues – including Jessica Armand as first author – report that tumor resistance depends on multiple factors (Armand et al., 2025).

The researchers, who are based at Columbia University and Emory University, studied hormone-driven and triple-negative breast cancer cell lines. All cells had an intact Rb/E2F pathway and were continually exposed to CDK4/6 inhibitors for over a month to induce drug resistance. After resistance emerged, some cells were taken off the treatment, while others continued to receive the inhibitors.

The results showed that cells kept on the treatment grew significantly more slowly than those taken off it, indicating that even resistant cells can remain partially sensitive to CDK4/6 inhibitors. More detailed analyses revealed that treated resistant cells stayed longer in the G1 phase and divided more slowly. They also showed reduced E2F activity and weaker Rb phosphorylation. With less phosphorylation, Rb remained active, and cells were halted in the G1 phase. Adding a CDK2 inhibitor reinforced this effect. Conversely, overexpressing a protein called Cyclin E, which works with CDK2 to push cells into the S phase even when CDK4/6 is blocked (Kim et al., 2025), weakened the inhibitor’s impact. These findings indicate that CDK2 helps drive the cell cycle forward even when CDK4/6 is compromised.

The study by Armand et al. changes how we think about drug resistance and suggests that resistant tumors might respond to treatment, but not as strongly as non-resistant tumors. Continuing treatment with CDK4/6 inhibitors could therefore help slow down cell division, especially when combined with CDK2 inhibitors. Rather than discontinuing treatment once resistance develops, reinforcing it may thus be a new clinical strategy. This may help explain why some trials have observed benefits from continuing CDK4/6 inhibitors (Kim et al., 2023). The results also highlight the need to target multiple points in the cell cycle: instead of focusing on a single enzyme, combination therapy may work better.

However, more work is needed to test the effectiveness and safety of this approach in patients. Tumors with mutations in the gene for Rb may not benefit from this strategy, and it is still unclear whether endocrine therapy may influence or modify responses to dual inhibition of CDK2 and CDK4/6. To identify which tumors will respond, more reliable biomarkers are needed (Zhang et al., 2023). Future research should explore how to personalize therapy based on each tumor’s biology.

Overall, these findings open new directions for both research and treatment. They highlight that even a modest slowdown of the cell cycle can have meaningful therapeutic effects, and in the context of drug resistance, delaying progression may offer a valuable advantage.
